# A hydraulic soft microgripper for biological studies

**DOI:** 10.1038/s41598-022-25713-1

**Published:** 2022-12-10

**Authors:** Sina Baghbani Kordmahale, Jian Qu, Anastasia Muliana, Jun Kameoka

**Affiliations:** 1 Department of Electrical Engineering and Computer Science, Texas A&M University, College Station, TX USA; 2 Department of Mechanical Engineering, Texas A&M University, College Station, TX USA; 3 Graduate School of Information, Production, and System Research, Waseda University, Kitakyushu, Japan

**Keywords:** Biomedical engineering, Electrical and electronic engineering, Mechanical engineering, Soft materials, Polymers, Engineering, Materials science, Nanoscience and technology, Techniques and instrumentation

## Abstract

We have developed a microscale hydraulic soft gripper and demonstrated the handling of an insect without damage. This gripper is built on Polydimethylsiloxane (PDMS) with the soft material casting technique to form three finger-like columns, which are placed on a circular membrane. The fingers have a length of 1.5 mm and a diameter of 300 µm each; the distance between the two fingers is 600 µm of center-to-center distance. A membrane as a 150 µm soft film is built on top of a cylindrical hollow space. Applying pressure to the interior space can bend the membrane. Bending the membrane causes the motion of opening/closing of the gripper, and as a result, the three fingers can grip an object or release it. The PDMS was characterized, and the experimental results were used later in Abaqus software to simulate the gripping motion. The range of deformation of the gripper was investigated by simulation and experiment. The result of the simulation agrees with the experiments. The maximum 543 µN force was measured for this microfluidic-compatible microgripper and it could lift a ball that weighs 168.4 mg and has a 0.5 mm diameter. Using this microgripper, an ant was manipulated successfully without any damage. Results showed fabricated device has great a potential as micro/bio manipulator.

## Introduction

The evolution in robotics in the past several decades opened new gripping technologies in various fields such as surgery, biological studies, and small object manipulation^1–6^. Minimally Invasive Surgery (MIS) is highly dependent on robotic technology to minimize patient trauma and improve clinical outcomes^7^. Occasionally, MIS still can cause complexities due to traumatic damages and more clinical uptake of MIS needs more flexible actuators to provide higher dexterity to the surgeon, minimize the blueprint, and have delicate gripper-tissue contact^7,8^. Also, handling biological specimen in various environments, and with different sizes need to be performed successfully by using robotic manipulators^9^. Most of the commercially available grippers with their rigid structures are not suitable to handle delicate and fragile biological objects and samples^9,10^.

To handle soft and fragile biological specimens, multiple grippers are proposed based on various actuation mechanisms such as shape memory alloys (SMA)^11^, piezoelectric^12^, electrostatic MEMS^13^, and various soft actuators^14^. Piezoelectric ceramics and thin films have been used extensively to develop various actuators that are proper to use in robots and end effectors^15^. The precise control of the movement of piezoelectric actuators, a high-power density, and a fast response time are the main benefits of piezoelectric actuators^16–18^. Piezoceramics can induce the development of various types of actuators, but still requires a complicated micro-displacement transmission mechanism, and assembling miniaturized actuators is challenging^19^. The high voltage required for piezoelectric material actuation is another drawback of this family of actuators that can delimit their applications in the fields of biostudy and surgery^20^.

SMA actuators use SMA wires or thin films as the actuating element. This simple mechanism can produce large force and stroke^21^. While various actuators can be developed with SMA wires and thin films, assembling them is more challenging when the size of the devices is smaller than a millimeter scale^22^. The complexity of the required heating/cooling systems alongside slow response are additionally delimiting drawbacks of the SMA actuators^23,24^. The proper and expensive thermal control system is essential^25,26^. The heating requirement for SMA elements can limit the application of these actuators to manipulate heat-sensitive biospecimen and live tissues due to lateral thermal damage^27,28^. Electrostatic MEMS actuators are mainly based on the silicon microfabrication process^29^. The well-established fabrication process made this group of actuators an ideal and cost-effective actuator as 2D structures and a good candidate for miniaturization^29,30^. The challenges in proper packaging and isolation of MEMS actuators are the main drawbacks of this family of actuators^31^. All aforementioned actuators are developed based on hard materials. This material feature is a drawback for handling fragile samples, and its solving will increase their complexities^32,33^. None of these actuators are satisfied to handle biological objects without damage. To extend to handle a fragile sample, a new level of gripper based on a proper combination of material and actuator should be developed.

Choosing the proper soft material and mechanism to fabricate a new gripper is essential for handling fragile biological samples^34^. Soft materials can be an alternative to hard polymers and metals, which using them allows grippers to mimic the properties of soft tissues and biological actuators, and provides robots with more flexibility^35,36^. To manipulate soft tissues, PDMS with Young’s modulus around 10^–6^ can be a better option in comparison to other soft polymeric materials and gels^37,38^. The development of soft grippers with Biocompatible PDMS, which has Young’s modulus similar to soft tissues, allows surgeons and researchers to delicately handle soft tissues and biological specimens^39–41^. Soft robots can be designed to actuate in response to different stimuli like pressure-driven, photo-responsive, thermally-responsive, magnetically-responsive, and electrically-responsive actuators^39^. Electrically actuated soft robots can be developed by using soft materials like cellulose. However, the developed centimeter-size actuators cannot be miniaturized, are sensitive to ambient humidity, have a slow response time, and need reliable encapsulation^42,43^. Magnetically responsive soft robots can be formed from a composite of polymers, gels, or other soft materials with magnetic fillers like magnetic particles. These soft robots can work in the encapsulated area and achieve a fast actuation (up to around 100 Hz). Still, these actuators need magnetic fields that require high energy consumption and big coils. Still, the areas that can be provided with proportionally strong fields are small and need complicated control systems^44,45^. Thermally actuated soft actuators are developed using soft gels as the soft actuator. While One of their main drawbacks is their slow response time, their functionality can be delimited to liquid environments. Lack of resistance to other stimulants, and unwanted motions make it difficult to handle fragile biospecimens. Another main problem of developed thermally actuated soft robots is their fabrication scalability^46,47^. Photo-responsive soft actuators can be used to develop soft grippers. Still, the slow response time is one of the main drawbacks of these soft robots. Using UV light to actuate them is another limiting factor to use in the peripheral area of biospecimen. Lack of thermal stability and the need for a straight sight between the light source and the actuator are other limiting factors^48,49^. Pressure-driven soft grippers have a fast response time and can produce an admirable amount of force, they are still faced with fabrication complexities, especially for smaller scales^50^. Pressure-driven actuators developed based on the deflection of the membrane have movement limitations and need additional mechanisms to increase the motion while this strategy can increase the fabrication cost and complexity^51,52^. Soft actuators developed based on balloon actuators contain deformable chambers and the deformation is usually governed by asymmetrical structure or heterogeneous material in the chamber’s structure^53,54^. This design is versatile but adds more fabrication complexities to small-scale grippers^55^. For instance, developing a micro-finger by using soft material mold casting and hermetic bonding have their complications because of molding difficulties and hermetic bonding flaws at the small feature sizes^56^. Forming a microgripper by using developed discrete micro fingers needs an extra complicated assembling step^57^. Flexible tubing connectors, as well as pressure-driven soft robots, eliminate constraints encountered by other soft microgripper developing approaches^55^; however, difficulties in the miniaturization and assembling of the micro-scale actuators require using expensive techniques^52,56^. After overcoming fabrication complexities, using a pressure differential mechanism in a soft actuator can help us to develop a low-cost microgripper with a high force production density^58^.

After determining the required amount of force, working media, response time, the required method of transferring force to the specimens, the favorite materials, and the suitable actuator, the proper microgripper can be developed^53^. In this paper, we have used low-cost 3D printing and soft material casting methods to fabricate monolithic PDMS-based three-finger-like columns on a deformable membrane to develop a microgripper. By developing this monolithic microgripper, we overcame the fabrication complexities of pressure-driven microgrippers. While we have used a low-cost fabrication method, we have avoided complicated assembling steps too. The finite-element method simulation was performed by using ABAQUS 6.12 software which can be a design tool for optimization microgripper designs. The bending characterization of the microgripper was investigated successfully by using optical microscope imaging and the experimental results agree with the simulations. The force production characterization was executed using AE-800 series piezoresistive microforce sensor and 543 µN was measured as the maximum force. Weightlifting ability was measured by gripping balls with various weights and diameters and 168.4 mg was measured as the maximum weightlifting ability of the gripper. To approve the ability of the developed device to manipulate fragile biospecimen, a live ant was gripped, held, and released successfully without any damage. The developed monolithic microgripper in this paper has used the benefits of pressure-driven actuators and PDMS while having low-cost and simple fabrication steps.

## Results

### Design and mechanism

**Figure 1:**
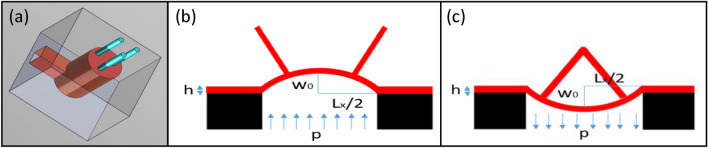
The designed microgripper with the fingers placed on the membrane and the opening/closing mechanism. (**a**) microgripper developed based on PDMS casting and is matched with microfluidics. The channel connected to the central void can be seen in this image. (**b**) Opening the fingers. (**c**) Closing the fingers.

The idea to achieve a 3D microhand-like device in this research is to form a micro-scale gripper that can close and open by adjusting the pressure in a cavity. The main part of the designed hydraulic 3D microgripper is created from a combination of a 150 µm PDMS-based membrane placed on the top of a cylindrical void and three columns, which are placed on the top of the membrane as fingers. The void, the membrane, and the three fingers form the monolithic gripper. The design of the microgripper that is compatible with microfluidics is pictured in Fig. 1a.

As it is depicted in Fig. 1, the fingers are placed on a deformable membrane. By deforming the membrane’s shape into concave or convex, the three fingertips on the top can cause opening or closing. Thus, the internal pressure of the cavity should be higher than the exterior’s pressure to create convex membranes and lower for concave membranes. The deformation of the membrane can be calculated, either analytically or numerically, based on the amount of pressure. The deformation mechanism based on this pressure difference is independent of membrane geometries. An approximate maximum displacement at the center of a circular membrane with the radius of a = L_X_/2 is given by Eq. (1)^59^.

1 $${W}_{0}\text{=} \frac{{P a}^{4}}{64{D}_{flex}}$$

This equation can give a very good insight into membrane deflection and show the effects of static pressure (P), dimensions and geometry (a), and material rigidity (D_flex_). By changing the membrane thickness, membrane diameter, or material stiffness, it is possible to have devices with different functional features. In Eq. (2), the deflection at (r) can be calculated^59^.

2 $$W\left(r\right)= \frac{P{a}^{4}}{64{D}_{flex}} (1-\frac{{r}^{2}}{{a}^{2}})$$

### Simulation and modelling

The finite-element software ABAQUS 6.12 was utilized to build a 3D model for simulating the hydraulic actuation of microgripper. A slightly compressible Gent hyperelastic free energy, having two parameters empirical constitutive model, is used for the hyperelastic volumetric and isochoric isotropic terms of the isotropic part of the constitutive model. The advantage of the model is to capture strain-stiffing at large strains, which are experimentally observed in soft materials. We separate the volumetric and deviatoric parts of the constitutive equation for the finite element implementation to avoid numerical problems such as element locking. ${W}_{V}(J)$ denotes a purely volumetric and ${W}_{D}(\overline{{I}_{1}})$ represents its deviatoric contribution represented by the slightly compressible Gent hyperelastic model given by,

3 $${W}_{D }(\overline{{I }_{1}} )=-\frac{\mu {J}_{m}}{2}\mathit{ln}(\frac{{J}_{m}- \overline{{I }_{1}}+3}{{J}_{m}})$$

4 $${W}_{v }\left(J\right)=-\frac{K}{2}\mathit{ln}\left(J\right)+ \frac{K}{2} \frac{{J}^{2}-1}{2}$$

$\mu$ is the shear modulus, ${J}_{m}$ is material constant, and $K$ is the bulk modulus. The uniaxial experimental data is fitted, and the material properties are $\mu =0.4\; \text{MPa}$, ${J}_{m}=5.5$ and ${\text{K}} = 2000$.

**Figure 2:**
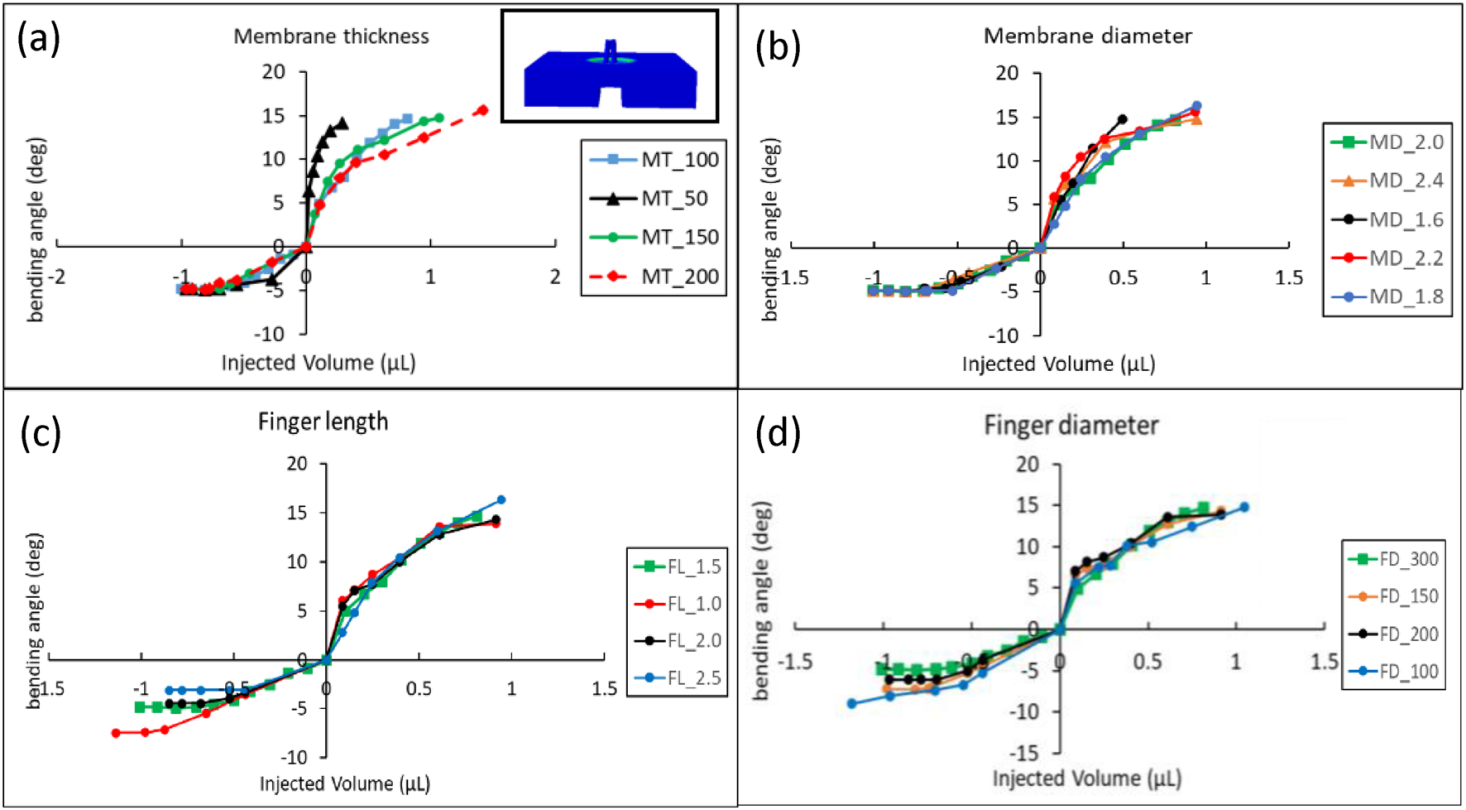
3D model and simulation result for a square-shaped microgripper and bending of fingers versus injected volume. (**a**) The deflection of the fingers versus injected volume can be seen here for membrane thicknesses of 50 µm, 100 µm, 150 µm, and 200 µm. (Inset)Simulation of deformation of the fingers in the closing regime. (**b**) The deflection of the fingers for various injected volumes can be seen in this figure for membrane diameters of 1.6 mm, 1.8 mm, 2 mm, 2.2 mm, and 2.4 mm. (**c**) The deflection of the fingers for various injected volumes can be seen in this figure for the length of the fingers of 1 mm, 1.5 mm, 2 mm, and 2.5 mm. (**d**) The deflection of the fingers for various injected volumes can be seen in this figure for diameters of the fingers of 100 µm, 150 µm, 200 µm, and 300 µm.

The deformation results from simulations are shown in Fig. 2. The displacement of the bottom surface of the model was constrained since the model was bonded horizontally on a plate of PDMS. Applied hydraulic pressures were set as pressure load which acts in the normal direction on the hydraulic channel. General surface-to-surface contacts were set up for the three fingers of the model in Fig. 2a (inset).

While the material parameters were obtained from the uniaxial experiment, to check the effects of changing various parameters on the deflection behavior of the microgrippers, the simulations of square-shaped microgripper were performed using various parameters. The thickness of the membranes, the diameter of the membranes, the diameter of the fingers, and the length of the fingers were the parameters that were used to study the devices. The simulations were performed for deflections of less than 15° in the opening regime.

In Fig. 2a, the deflection of the fingers versus the variation of injected volume for various thicknesses of the membrane can be seen. As the membrane gets thicker, it requires more force to deform. Also, a thicker membrane results in a slower microgripper that needs a bigger injected volume to bend at the same angle in comparison with a microgripper with a thinner membrane.

In Fig. 2b, the deflection of the fingers versus the variation of injected volume for various diameters of the membrane can be seen. There is no obvious difference between the behavior of microgrippers with various membrane diameters. Still, the membrane’s diameter can affect the overall size of the microgripper device. the smaller the diameter of the membrane, the smaller the overall size of the microgripper. In Fig. 2c, the deflection of the fingers versus the variation of injected volume for various lengths of the fingers can be seen. The length of the fingers doesn’t affect the bending angle in the opening regime. Still, the results of simulations show that shorter fingers can cause a higher bending angle. In Fig. 2d, the deflection of the fingers versus the variation of injected volume for various diameters of the fingers can be seen. The thickness of the fingers cannot affect the deflection angle in the opening regime. Still, it limits the closing angel when the fingers are thicker.

To choose the proper parameters for the required microgrippers, alongside the application, the fabrication feasibility based on the fabrication method should be considered. For instance, to choose the diameter of each finger, the possibility to prepare the required molds for the soft material molding method is the main parameter. When the center-to-center distance of fingers is fixed at 600 µm to provide a proper area to grab an object with the required size, the diameter of the fingers is set to be 300 µm, which is the smallest diameter that can be repeatedly fabricated based-on soft material molding with the available 3D printer. The lengths of the fingers are 1.5 mm for a microfluidic-compatible device. This length was chosen based on the required size of the device, finger movement and controllability, and produced force. The longer the finger, formed the bigger device and caused a smaller transfer of force to the specimen. The diameter of the membrane affects the size of the device. The diameter of the membrane is 2 mm for the designed microgripper. The device with a smaller membrane diameter has a smaller whole device size. The thickness of the membrane is another parameter in designing the microgrippers. While a thinner membrane results in a faster microgripper that can bend with a smaller injected volume of water or a smaller hydraulic pressure, a thicker membrane will cause a slower device that needs a bigger hydraulic pressure or a bigger volume of injected water to achieve the same amount of bending of fingers. Because of this 150 µm was chosen as the thickness of the membranes to develop the required microgrippers. This amount was chosen rather than 50 µm and 100 µm of membrane thickness to avoid a fast movement of the fingers versus injected volume of water and was chosen rather than 200 µm of the thickness of the membrane to avoid a required larger volume of injected water to actuate the device that decreases the response time of the grippers.

### Fabrication

**Figure 3:**
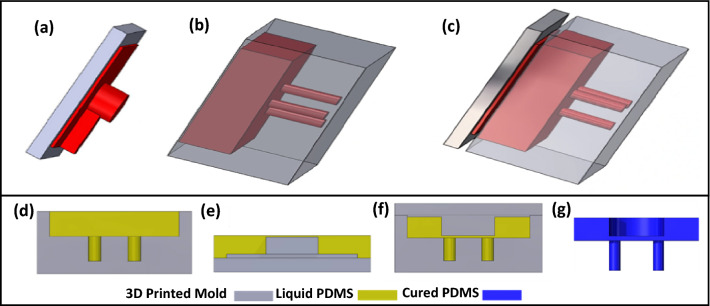
The schematic model for 3D printed molds and microgripper fabrication steps (**a**,**b**) The mold cap and mold for microgripper. (**c**) The closing main part and cap of the mold to shape the whole mold for the microgripper. The red color shows the internal surfaces of the molds. These surfaces will be in touch with the liquid PDMS and will shape microgrippers. (**d**,**e**) The 3D printed mold was filled with and de-bubbled liquid PDMS, and the mold cap was covered with the same PDMS. (**f**) Closing the mold and the mold cap. The liquid and de-bubbled PDMS were encapsulated in the 3D printed parts. As it can be seen, in this step the parts shown in (**d**) and (**e**) that filled and covered with PDMS are closed together to form the encapsulated area. (**g**) PDMS was cured at 25 °C in 1 week. The cured PDMS was removed from the 3D printed molds and the functional part of the microgripper was formed.

The microgripper is fabricated from polydimethylsiloxane (PDMS) through low-cost soft material molding^60–62^. The required molds for this process were fabricated using a stereolithography 3D printer. To finalize the fabrication of 3D printed molds, they were exposed to light curing, thermal curing for 72 h at 65 °C in the oven, and silane gas as the surface treatment, respectively. Silanization can make the surface more hydrophobic through the inclusion of silane gas^63,64^. The parts of the 3D printed mold are illustrated in Fig. 3. In Fig. 3a,b the mold elements for the device are displayed. The first part of the mold (shown in Fig. 3a) was responsible for forming the central void as a cavity and the membrane where the fingers are placed, while the second part of the mold (shown in Fig. 3b) was designed with three holes to form the three fingers. The two parts of the mold can be attached to form an encapsulated closed volume as a complete mold. In Fig. 3c the whole mold is shown for microgripper fabrication. For PDMS the polymer and initiator were mixed with the (15:1) ratio to achieve a very soft and deformable material. The curing of PDMS was executed in 1 week at 25 °C. The combination of the hydrophobic surface of the molds and a very flexible soft material made it possible to remove the cured PDMS from the molds.

**Figure 4:**
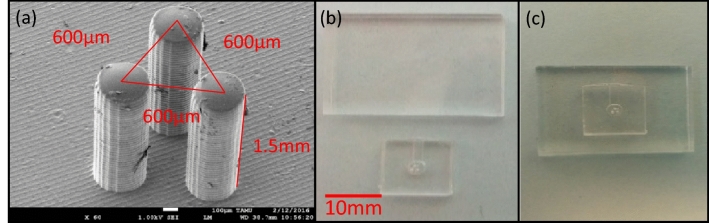
SEM image of three fingers, and assembling and finalizing the microgripper. (**a**) SEM image of fingers. The diameter of each finger is 300 μm and the center-to-center distance between every two fingers is 600 μm. The Triangle in the image shows the center-to-center distances between fingers. The length of each finger is 1.5 mm. (**b**) Microgripper’s elements. (**c**) Finalized microgripper.

The fabrication steps for the microgripper are pictured in Fig. 3. To form the devices, the liquid PDMS was poured in the main part of the molds, which contains three holes. Also, the mold’s cap, which was responsible to form the channel and cavity, was covered with liquid PDMS (Fig. 3d,e). Then they were placed in a vacuum to remove all the bubbles from the liquid. The two parts of the molds were closed (Fig. 3f). Finally, the molds with encapsulated liquid PDMS inside of them was placed in the vacuum to remove all the residual entrapped bubbles. After leaving the molds for 1 week at room temperature (25 °C), the two pieces opened, and the cured PDMS was removed slowly (Fig. 3g). Because the cured PDMS was very soft with 1 MPa young modulus, it was possible to remove it from the molds without damage. The SEM image of the three fingers of the microgripper is shown in Fig. 4.

The final step of the fabrication was to assemble the microgripper’s parts and finalize the device. For this purpose, the previously fabricated structure was bonded on a plate of cured PDMS by using oxygen plasma treatment and water as the bonding liquid^63,64^. When the water evaporates eventually, the strong hermetic bond formed between two pieces of cured PDMS, and the whole cavity and device formed successfully. The parts and finalized device are shown in Fig. 4.

### Characterization

#### Deflection characterization

**Figure 5:**
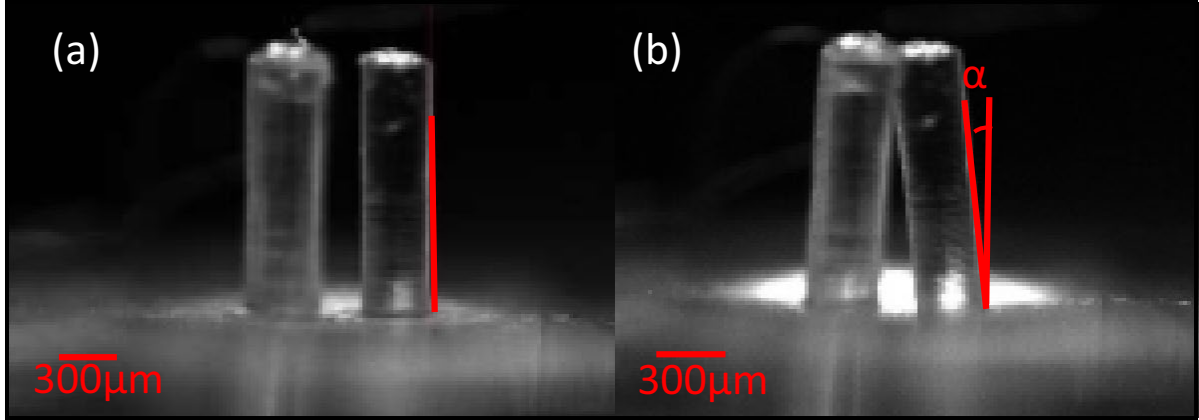
The side view of fingers, and the side view of bending fingers and bending’s angle. (**a**) The side view of the microgripper’s fingers. (**b**) The side view of the bending finger and the measured angle of bending (α).

The deflection characterization of the microgripper was performed using precise microliter syringes under an optical microscope. To characterize the degrees of deflections, the device was placed under an optical Nikon microscope. The deflection-volume graphs were obtained by changing the injected water’s volume into the microgripper’s cavity. To precisely control the injected volume of water in the cavity, precise micro-syringes, fabricated by Hamilton, were used. After manually injecting of required volume of water into the cavity by using the micro syringe and through proper tubing, the deflections were measured on optical images that were taken by using the software of Nikon microscope. The side view of the bent fingers and the measured angle are depicted in Fig. 5.

**Figure 6:**
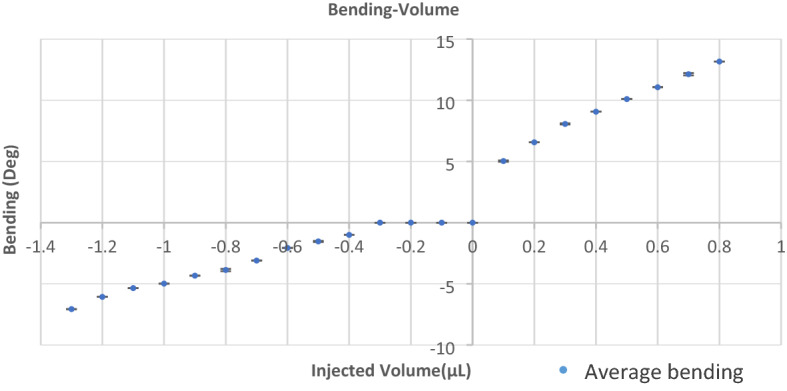
The microgripper bending characterization. The bending of the finger was measured under an optical microscope. The angle (α) was measured as a function of the injected volume of water in the cavity. The negative angles are representing the angles during the closing regime.

As it is obvious in Fig. 5b, a micro-cage was formed between all three fingers after the full closing of microgripper. This micro-cage can be useful for grabbing small and fragile objects. The results of bending-injected volume experimental measurements for the microgripper are shown in Fig. 6.

**Figure 7:**
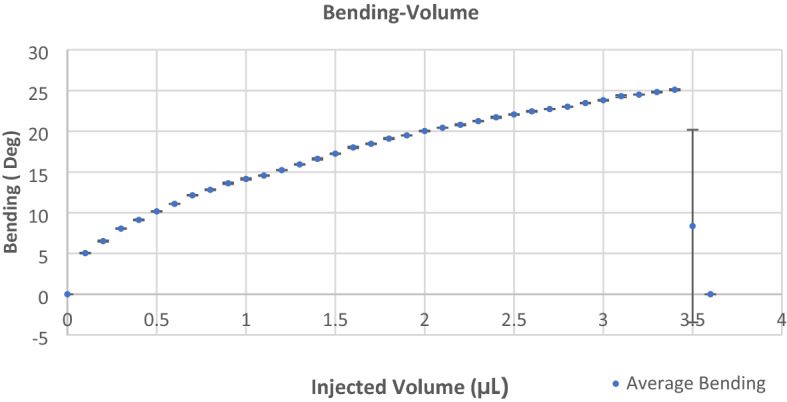
Non-linear behavior of the microgripper and rapture of the membrane. The bending of the finger was measured under an optical microscope. The angle (α) was measured as a function of the injected volume of water in the cavity. This measurement was performed for higher injected volumes and the rapture of the membrane was observed for volumes higher than 3.5 (µL) and angles higher than 25°.

The experiments were performed to observe deflections less than 15°. After performing deflection characterization or grabbing a specimen that caused more than 100 times of bending the membrane, no rapture in the membrane, hysteresis in the deflection curves, or shift in the performance was observed. Still, deflections of more than 25° can cause rapture of the membrane, leakage, and deterioration in the microgripper’s performance. In Fig. 7, the immediate rapture of the membrane and closing of the microgripper for higher volumes, and the nonlinear behavior of the microgripper can be seen.

#### Force characterization

**Figure 8:**
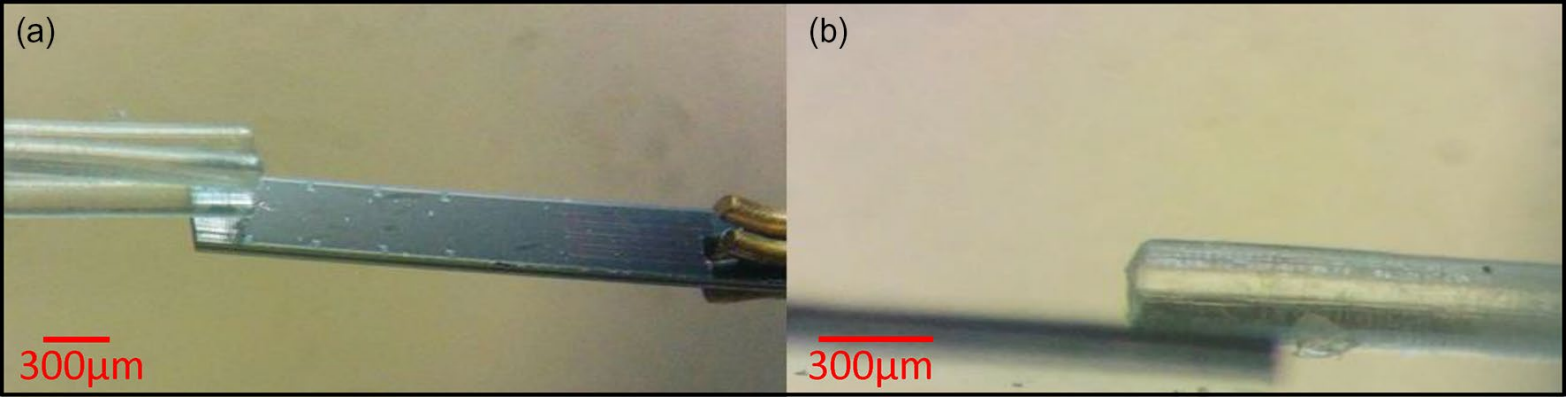
Force measurement by using an AE-800 sensor. (**a**) An optical image of the microgripper and the force sensor. The suggested placement of the microgripper and the sensor can be seen. (**b**) The side view of a finger and the cantilever of the sensor.

To measure the produced forces of the microgripper, an AE-800 microforce sensor was used. This sensor works based on a piezoresistive mechanism. For effective measurement to avoid any unwanted misplacement during the force measurement, the sensor and the microgripper were placed under a stereoscope. To ensure the proper measurement, the gap between the finger and the sensor’s cantilever should be zero. Also, the spatial overlap between the finger and the cantilever was adjusted precisely to a specific 300 µm amount for all the experiments. In Fig. 8, the optical images of the finger and the sensor can be seen.

**Figure 9:**
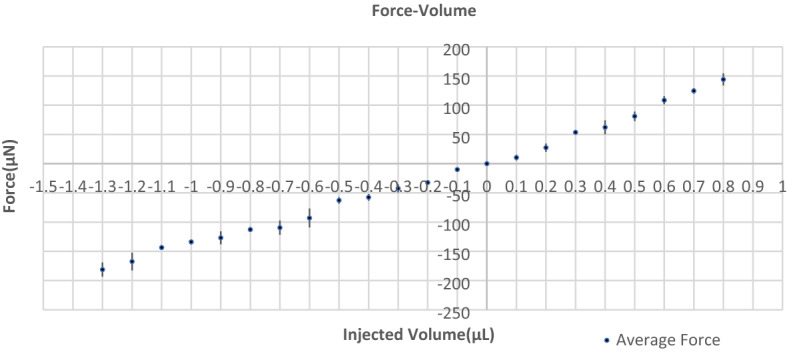
Force measurement. The force produced by one finger of the microgripper is measured under an optical microscope by using an AE-800 piezoresistive force sensor. The produced force changes are shown versus the changes in the injected volume of water in the cavity. The negative forces are representing the produced forces during the closing regime.

The result of the force-injected volume measurements for the microgripper is shown in Fig. 9.

The produced hydraulic force by injecting water into the chamber was transferred to the membrane, and then from the membrane to the fingers. This force can be transferred to the object to grab it. The transferred force to the object is limited because of the compressibility and bendability of soft PDMS. Fingers can be bent, and this delimited the transferred force to the object. Also, the membrane is a thin 150 µm layer of soft PDMS that can be bent and delimit the transferred force. To increase the load capacity of the developed microgrippers, the developer can change some parameters on the fingers, the membrane, and the material. Soft material with a higher Young’s modulus and less compressibility can reduce the compression and unwanted bending of the fingers, and increase the load produced by the gripper. At the same time, a higher Yong’s modulus can make it difficult to fabricate the devices with the same encapsulated soft material molding. A shorter and thicker finger can increase the load, while a longer and thinner decrease it. A thicker membrane can reduce the local bending of the membrane on the connection points of fingers and the membrane, which can increase the produced load of grippers.

### Comparison of simulation and measurements

**Figure 10:**
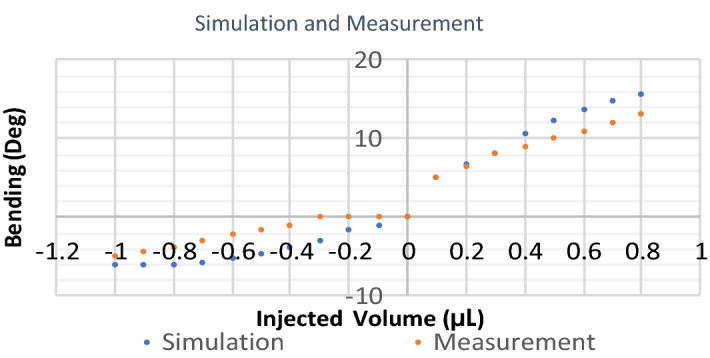
Comparison of the simulation and experimental measurement. The comparison between simulation and measurement for the microgripper. The simulation and experimental measurement show the same trend and almost the same slope as depicted in the figure.

The comparisons of the simulation and experimental measurements are depicted in Fig. 10. This graph shows that the simulation and experimental results are in very good agreement, which means the 3D model is appropriately developed and can later be used in the design process of the new devices. The simulation and experimental measurement have the same trend and show almost the same slope as depicted in Fig. 10. The Slope in some pieces is exactly similar, but in the opening regime, the simulation shows an overall slope of 15.71 and the measurements show an overall slope of 11.43. Similarly in the closing regime, the simulation shows an overall slope of 8.57 and the experiment shows 7.14 for the same parameter. While the point-to-point values can be different, the same trend and close slops show the agreement between the behavior of the device in the simulation and experiment. The differences between simulation and measurements can be because of leakage, the uncertainty of the soft material casting method, and the un-uniformity of the material. Also, the model may need some improvement. Further development of this simulation can provide a powerful tool for the design and optimization of the parameters of required microgrippers in the future.

### Biospecimen manipulation

Gripping and manipulation of fragile biological specimens can be challenging due to potential damaging may happen during this process to the specimen. This damage can be the result of chemical shocks, thermal shocks, exceeding pressure, electrical discharge, etc.^65,66^. The developed microgripper is made from a biocompatible soft material and the stimulant for the actuation of this microgripper is hydraulic pressure. In the absence of any other hazardous parameter to the tissues or a live specimen, an extra force can be the only reason to damage cells, tissues, and all types of a live specimens. In the case of an ant gripping, rapture of the body, and damage to the neck and joints can be among the damages that may be happened during the gripping process and because of an extra force^67^.

**Figure. 11:**
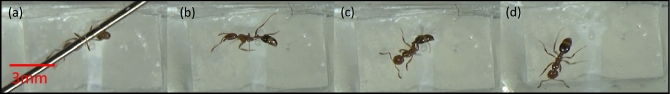
Manipulation of a live insect. (**a**) Start the grabbing of the ant. A needle is used to hold the insect on the top of the gripper. (**b**) Successful handling of a live ant by using a square-shaped device. (**c**) Opening the gripper to release the ant. (**d**) Ant left the gripper without any damage.

To prove the hypothesis that the developed microgripper can manipulate a biological fragile sample precisely, the manipulation of an ant was performed under a microscope. The length of the ant was almost 3 mm, and the average width of its body was around 400 µm. Microgripper was able to grab, hold, and release a live ant without physically damaging it. So, by adjusting the pressure and the finger location, the manipulation of the fragile specimen was performed successfully. The manipulation of an ant is shown in Fig. 11. For this purpose, 543 µN of force was induced to the ant, which was the maximum measured force the device produced at the onset of the fingers' full closing. Also, by sucking more water from the cavity no damage to the insect was observed.

### Maximum grasping ability

**Figure 12:**
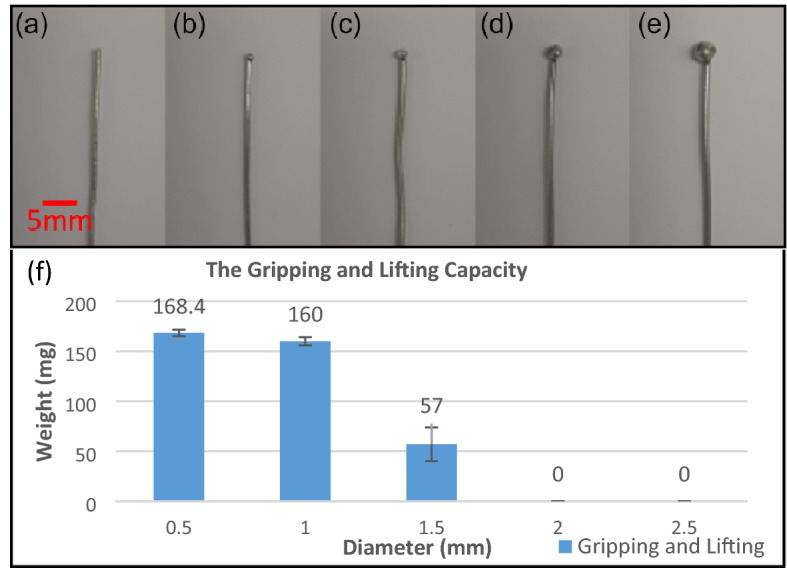
Balls with various sizes and adjustable weights, and weightlifting ability. The size of the balls is adjusted by melting the tip of the solder wire. (**a**) 0.5 mm, (**b**) 1 mm, (**c**) 1.5 mm, (**d**) 2 mm, (**e**) 2.5 mm. (**f**) The maximum weight that a developed microgripper can lift for a ball with various diameters can be seen here. The microgripper cannot grip and lift a ball of 2 mm in diameter or more.

While the ability of the developed microgripper to grip and handle a small object depends on several factors like size, overall shape, surface morphology, and weight of the object, it is useful to calculate the opening values of the gripper. In the case that the object has a spherical shape if the object is grabbed in the middle of the fingers, the diameter of the sphere can be up to 1.43 mm. If the same spheres are light enough to be grabbed by the tip of the fingers, their diameter can be up to 2.86 mm. Because the fingers can be closed completely, theoretically there is no minimum size these grippers can grab and handle. This ability of the gripper can be measured experimentally by lifting ballas with different diameters and weights. For this purpose, various balls were made from solder wire by heating one tip of the solder wire with a specific length and weight. The prepared weights with different ball-tip diameters can be seen in Fig. 12. The benefit of this technique is the possibility of adjusting the total weight by changing the length of the wires, while the size of the ball (tip of the wire) is constant.

We examined the capacity of the microgripper to manipulate various sizes and weights, by grasping and lifting various diameters of the balls, and the results can be seen in Fig. 12f. The maximum weight the microgripper can lift is 168 mg for 0.5 mm of diameter.

## Discussion

The innovative microgripper was developed by using soft material molding technique and with the use of 3D printed molds. This monolithic microgripper made from PDMS, with their produced force and motion precisely controllable, made this innovative manipulator a practical option for biospecimen handling.

As mentioned in the fabrication part, the proposed microgripper can be fabricated in a single-step molding of PDMS. The innovative, encapsulated, molding technique was used to fabricate the microgripper introduced in this paper. The actuation mechanism is the bending of a flexible membrane due to pressure differences on two surfaces of the membrane. As it can be concluded from Eq. (1) the higher-pressure difference, or a thinner membrane will cause more deformation. This is also approved by simulations. At the same time, a thicker membrane or a stiffer material can limit the deformation of the membrane when exposed to the same pressure difference or with the same amount of injected water volume. The fingers of the microgripper were placed on this flexible membrane.

The precision in the bending of the fingers is revealed from the characterization. This is because the bending of the fingers is proportional to the precisely injected volume into the device that was controlled by precise micro-syringes. The wide range of the precise bending of the fingers, alongside the limitation of the transferred force to the object because of using soft material, confirms the ability of the microgripper to manipulate a wide range of fragile objects. The bending angle for each finger ranged from 8° of closing to 13° of opening for the device. The precision of the fingers’ deflection highly depends on the microliter syringes’ precision and the precision in controlling the syringes. Each step of the injected volume into the cavity of a microgripper was 0.1 µL in this study. And the minimum step of the measured bending angle was 0.5° for the device.

The force measurement revealed the maximum produced force of the microgripper is smaller than 1 mN, which means it is suitable for biospecimen handling^68^. The maximum absolute produced force of a single finger in a device is around 181 µN, which means three fingers can impose around 543 µN on a specimen. The force-volume ratio, which can be described as the absolute force divided by the volume of the gripper and actuator, for the microgripper is 1.13 mN/mm^3^. As references, other soft grippers have a maximum absolute produced force of 50mN, 3mN, and 2.2 mN, and when the size of the grippers take into account the force-volume ratio is 0.78, 3.3, and 0.047 mN/mm^3^ respectively^69–71^. Both the produced force and force-volume ratio are higher or comparable with other soft manipulators in the literature^57^. Also, the ability of this developed device to lift various weights was measured by lifting spheres with various weights and diameters. 168.4 mg for 0.5 mm spheres was the maximum weightlifting capacity of the device.

The simulation of the microgripper revealed that the 3D model in the finite-element simulation can be used successfully to predict the actuation of the fingers. The results of this simulation can be used as a design tool to optimize the required parameters of the device. Disagreements between the simulation and experimental measurements can be mainly because of microscale variation of used PDMS in the membrane, soft material variations during the fabrication, or fabrication method resolutions. Absorption of the hydraulic pressure by different parts like soft tubes that were used for transfer pressure from the syringes to the device or soft surfaces except the membrane can be other issues that cause differences between simulations and experimental measurements. While more refined models can cause better prediction of microgripper moving manner, the current simulation shows a proper prediction of the deflection of the fingers.

By using 3D printers with higher resolutions, more levels of miniaturization can be achieved^72^. Also, integration of the micro/nano sensors into the developed microgrippers can pave the way to develop the micromanipulators with the ability to diagnose and detect during the performing of required procedures^73^.

## Methods

### Microgripper fabrication

The fabrication process was started by 3D printing the proper molds by using Perfactory Microprinter made by EnvisionTEC. After Printing the molds, the light-curing of the molds was done. And they are left in the oven at 65 °C for 72 h. After that, the molds were covered by silane gas, because the untreated molds are stickier for PDMS. As depicted in Fig. 3, the molds are combined from two different pieces and they will make an enclosed space after assembling. So, for fabricating PDMS-based structure, the molds first should be filled and covered with PDMS and remove the bobbles in a vacuum, then the molds will be closed, and finally, they will be sunk in PDMS and again place in the vacuum to remove the potentially remaining bobbles for 30 min. The molds and PDMS were left at room temperature (25 °C) for 1 week and then the slowly cured PDMS-based structures were removed carefully from the molds. To shape up the final structure, the PDMS-based structure had been bonded to the surface of a sheet from the same material, and finally, a piece of a tube was connected to the channel to make the device operational.

### Simulation

As mentioned, ABAQUS 6.12 was used to build up a 3D model for simulating the hydraulic actuation of the microgripper. The model used 376,938 tetrahedral elements and consisted of 10-node modified hybrid tetrahedral elements (ABAQUS C3D10MH). Modified tetrahedral elements provided a good convergence rate and prevented volumetric locking. Hyperelastic material models were highly capable of describing the nonlinear response of complex materials. With the experiment’s data fitted and analyzed, the PDMS was modeled as an incompressible and isotropic Gent model, which was satisfied with convergence and solution stability. The Gent model with the isotropic condition was applied and the material properties were $\mu = 0.4\; \text{MPa}$ and $J_{m} = 5.5$ for the studied PDMS material.

### Opening and closing characterization

For characterization, the opening and closing of the microgrippers, and achieving the “Deflection-Injected Volume” curve, the microgripper was placed under an optical microscope and was connected to a microliter precise syringe. The microliter syringes used in this study were fabricated by Hamilton. The injection of proper volume was performed manually with these micro-syringes. By changing the injected volume in the cavity and measuring the finger’s deflection under the microscope the characterization was performed successfully and the deflection-injected volume curve was achieved.

### Force measurement

AE-800 microforce sensor is a cantilever-like piezoresistive one used to measure the force produced by the fingers of the microgripper. For proper placement of the finger over the sensor, the whole process was performed under an optical stereoscope.

### Biospecimen manipulation

An ant was chosen as a biospecimen in this project, grabbed, and manipulated successfully as is shown in Fig. 11. For this purpose, the ant was placed on an ice pack and halted due to low temperature. This made it easier to grab the insect. The grabbing of the biospecimen was performed under a Nikon Stereoscope. After grabbing the ant, it was separated from the ice pack in a way it warmed up and started to move again. After 1 min of holding the moving insect, it was released, and no injuries were observed.

### Maximum grasping ability

To check the capacity of the developed microgripper to grab and lift objects with various weights and sizes, the balls with various diameters were prepared by melting a tip of soldering wires. The length of the attached wire to the ball was adjusting the overall weight of the object. Then the balls were grabbed and lifted vertically to measure the maximum weight the gripper can lift for each diameter.

## Data Availability

The datasets used and/or analyzed during the current study available from the corresponding author on reasonable request.
